# Utility of Urinary miRNA Biomarkers for Canine Urothelial Carcinoma Diagnostics

**DOI:** 10.3390/vetsci12070621

**Published:** 2025-06-27

**Authors:** Alexandra Kehl, Heike Aupperle-Lellbach, Maria Brockmann, Anna-Lena van de Weyer, Marielle Appenzeller, Katja Steiger

**Affiliations:** 1 LABOKLIN GmbH&CO.KG, 97768 Bad Kissingen, Germany; aupperle@laboklin.com (H.A.-L.); brockmann@laboklin.com (M.B.); vdweyer@laboklin.com (A.-L.v.d.W.); appenzeller@laboklin.com (M.A.); 2 School of Medicine, Institute of Pathology, Technical University of Munich, 81675 München, Germany; katja.steiger@tum.de

**Keywords:** miRNA, dog, urothelial carcinoma, urine, biomarker, cystitis

## Abstract

Urothelial carcinoma (UC) is one of the most frequent tumors in dogs. Besides cytology, histology, and testing for a *BRAF* mutation, non-invasive biomarkers would benefit the early detection and therapy of UC. This study aimed to compare the detectability of miRNAs in urine sediment and supernatant and to assess their potential as biomarkers for UC. The study involved two phases with 47 canine samples in total; in a pilot trial, ten different miRNAs (miR-16, 21, 103b, 106b, 146, 155, 182, 221, 222, and 375) were isolated from the urine sediments and supernatants from seven healthy control dogs and seven dogs with UC. In a further step, eight miRNAs were isolated from urine sediments from 18 healthy dogs, 11 dogs with purulent cystitis, and 18 dogs with UC. The detectability of miRNAs was improved when isolated from the urine sediment compared with the supernatant. MiR-16 was not deregulated, and miR-106b showed significantly lower expression in cystitis compared with the control. Lower copy numbers were seen for miR-21, 182, 221, and 222 in cystitis cases compared with the controls and UC, respectively. Deregulation was observed for miR-155 and miR-375 between all three groups. A panel including miR-182, 221, 222, 155, and 375 has the potential to discriminate among all three groups in a two-step approach.

## Simple Summary

Urothelial carcinoma (UC) is a common cancer in dogs, and detection methods beyond cytology, histology, and testing for a *BRAF* mutation are needed. MiRNAs are short RNAs that do not code for a protein but regulate the expression of genes. This study tested specific microRNAs (miR-16, 21, 103b, 106b, 146, 155, 182, 221, 222, and 375) in canine urine to see if they could serve as non-invasive biomarkers. The results showed that miRNAs are easier to detect in urine sediment than in the liquid part (supernatant). Certain miRNAs showed differences between healthy dogs, those with bladder inflammation (cystitis), and those with urothelial cancer. A group of five miRNAs (miR-182, 221, 222, 155, and 375) may help to distinguish between these three conditions.

## 1. Introduction

Urothelial carcinoma (UC) is one of dogs’ most common tumors, representing about 2% of all canine cancer cases [1]. Some breeds, like Terriers and Shetland Sheepdogs, show a predisposition for this tumor [2]. The diagnosis is usually performed invasively by a biopsy and cytology and, in most cases, after the dog has been showing signs such as hematuria, stranguria, and pollakiuria, which can be misinterpreted as signs of cystitis [3]. Non-invasive tests for early detection and/or screening by testing the *BRAF* V595E variant and/or copy number alterations are commercially available [4,5,6], but UCs without these genetic alterations are not detected with these methods, and, therefore, the sensitivity is about 64–70% [6]. Additional biomarkers for the early detection of all UCs, independent of the presence of known genetic alterations, would be of benefit for the affected dogs.

MiRNAs are small non-coding RNAs that regulate the expression of genes by translational suppression or enhancement and are involved in cell development and apoptosis [7]. Furthermore, miRNAs seem to play an essential role in oncogenesis by having tumor-suppressive or oncogenic effects [8,9,10]. MiRNAs are released in the bloodstream or other body fluids by cells actively or after apoptosis, where the miRNAs are stabilized by binding proteins or packaged in exosomes and microvesicles [11]. Therefore, they have been receiving attention as biomarkers for cancer in humans [12] and dogs, for example, in lymphoma and mammary tumors [13,14,15].

Two studies have examined altered miRNA profiles in canine bladder tissue affected by UC [16,17]. Both studies identified several differentially (up- or down-) expressed (DE) miRNAs in canine UC tissue, but the specific miRNAs differed between the studies. Further qPCR analysis in urine samples revealed significantly lower levels of miR-103b and miR-16 in dogs with UC compared to those with other lower urinary tract diseases [18]. Another study found higher levels of miR-182, 221, and 222 in extracellular vesicles (EVs) of urine samples of dogs with UC compared to healthy or cystitis samples that were not further characterized [19].

In humans, multiple studies investigated miRNAs as potential biomarkers in urine. They found several DE miRNAs, with some appearing several times: miR-21, 146, 155, and 375 were described as being up-regulated in urinary cells as well as in urine supernatants and EVs [20,21,22,23,24,25,26,27,28,29,30]. These have not been investigated or reported in dogs so far [31].

The aims of the present study were as follows: (1) to assess the best sample material for measuring miRNA in urine by comparing supernatants with sediments; (2) to identify potential miRNA biomarkers for canine UC by comparing promising canine and human miRNAs in normal urine samples, cystitis, and carcinoma samples.

## 2. Materials and Methods

### 2.1. Dogs and Sampling

**Figure 1:**
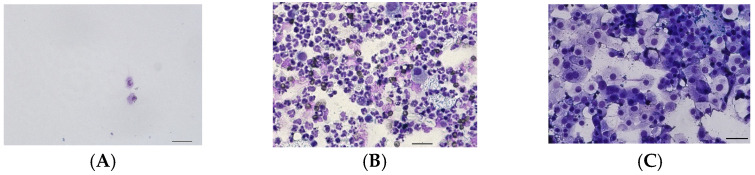
Examples of the cytological pictures from urine sediments of the groups: cell-poor control (**A**), severe purulent cystitis with bacteria (**B**), and urothelial carcinoma (**C**) (bar = 50 µm).

Urine samples (*n* = 47) were submitted to LABOKLIN GmbH & Co. KG, Bad Kissingen, Germany, for routine diagnostic examinations (urine status, *BRAF* mutation analysis). All urine samples were stored at −80 °C after the day of sampling until RNA isolation (7–28 days). Cytological examination of Diff Quick-stained slides was performed on all samples (Figure 1). Details regarding the breed, sex, and age of the dogs sampled can be found in Table 1.

Urine from dogs that showed no significant changes in the macroscopic, physical, chemical, or cytological examination was used as a control group (*n* = 18). Three control dogs were presented due to incontinence but were otherwise unremarkable in the general examination, according to the submitting veterinarian. Five dogs were presented for a follow-up examination after an episode of cystitis or crystalluria or for a routine check-up (“check-up” examination). According to the treating veterinarian, all of them were clinically unremarkable (at the time of sampling). The cytological investigation of all control dogs was negative for inflammation or neoplastic cells (Figure 1A).

The dogs in the cystitis group (*n* = 11) showed moderate-to-high numbers of degenerated neutrophils and occasionally bacteria and erythrocytes in cytology (Figure 1B). These findings corresponded to the referring veterinarian that these dogs were presented with clinical signs of cystitis.

Cases with UC (*n* = 18) were derived from routine analyses of the *BRAF* V595E variant, since this variant’s presence in urine indicates UC [4,6]. Cytologically, besides atypical urothelial cells, some erythrocytes and minimal numbers of neutrophils were seen (Figure 1C). UC was diagnosed by *BRAF* testing and cytological examination.

According to the terms and conditions of LABOKLIN and the decision of the government of Lower Franconia RUF-55.2.2-2532-1-86-5, no special permission has to be obtained from the animal owners or the animal welfare commission for examinations on the residual samples that are not needed for any further diagnostics.

### 2.2. RNA Isolation

The total miRNA was isolated from 200 µL of serum supernatant using the miRNeasy Serum/Plasma Advanced Kit (QIAGEN, #217204, Hilden, Germany), according to the manufacturer’s instructions (miRNeasy Serum/Plasma Advanced Kit Handbook, Version April 2021). As a spike-in control, cel-miR-39 was added. The total miRNA was eluted in 40 µL of RNase-free water.

A total of 1 mL of urine was centrifuged at 250 g for 5 min to pellet cells. The total miRNA was isolated from the pellet using the miRNeasy Tissue/Cells Advanced Kit (QIAGEN, #217684, Hilden, Germany), according to the manufacturer’s instructions (miRNeasy Tissue/Cells Advanced Kit Handbook, Version February 2021).

### 2.3. Quantification of miRNA by ddPCR

The quantification was carried out by ddPCR using specific microRNA assays (Thermo Fisher Scientific, #4427975, Waltham, MA, USA). Reverse transcription for TaqMan^TM^ miRNA assays was performed using the TaqMan^TM^ MicroRNA Reverse Transcription Kit (Thermo Fisher Scientific, #4366597, Waltham, MA, USA), according to the manufacturer’s instructions, with a specific primer from the TaqMan^TM^ miRNA assay (Thermo Fisher Scientific, #4427975, Waltham, MA, USA). According to the manufacturer’s instructions, reverse transcription for Advanced TaqMan^TM^ miRNA assays was performed using the TaqMan^TM^ Advanced miRNA cDNA Synthesis Kit (Thermo Fisher Scientific, #2873256, Waltham, MA, USA). The ddPCR was run on a QX200 Droplet Digital System (Bio-Rad, Hercules, CA, USA) using a ddPCR supermix for probes (Bio-Rad, Hercules, CA, USA) and a specific TaqMan^TM^ (Advanced) miRNA assay (Thermo Fisher Scientific, #4427975, Waltham, MA, USA).

Different miRNAs described as deregulated in the urine of human and canine UC cases were screened for a specific commercially available miRNA assay. TaqMan^TM^ (Advanced) miRNA assays were tested for their functionality in ddPCR. Finally, the following targets were chosen: miR-16 (Thermo Fisher Scientific, assay ID 477860_mir), miR-103b (assay ID 478621_mir), miR-106b (assay ID 478412_mir), miR-221 (assay ID 477981_mir), miR-182 (assay ID 477935_mir), miR-222 (assay ID 477982_mir), miR-21 (assay ID rno481342_mir), miR-146 (assay ID 481344_mir), miR-155 (assay ID 477927_mir), and miR-375 (assay ID 478074_mir). The exogenous miRNA cel-miR-39 (assay ID 000200) was used for normalization of isolation and amplification of the cell-free miRNA from urine supernatants [32], whereby endogenous RNU6B (assay ID 001973) was used to normalize the cell numbers in urine sediments [18].

An amount of 10 µL of cDNA was added to 11 µL of ddPCR supermix and 1 µL of TaqMan^TM^ miRNA assay. The generation of droplets was carried out in 8-well cartridges: 20 µL of the above-mentioned mix of cDNA, ddPCR supermix, and miRNA assay was added to one well, and 70 µL of oil was added to the designated well. The droplets were generated with a Droplet Maker (Bio-Rad, Hercules, CA, USA) and transferred to a 96-well plate. After sealing the plate, PCR was run with 35 cycles of 94 °C for 30 s, 60 °C for 30 s, and 72 °C for 30 s. The measurements were carried out using the QX200 Droplet Reader and the QuantaSoft software Version 1.7.4.0917 (Bio-Rad, Hercules, CA, USA). Each PCR was duplicated; the mean values were used for further processing. Normalization was performed with spike-in control cel-miR-39 and RNU6B, respectively [18,32].

### 2.4. Statistical Analysis

The normalized miRNA values of the samples from the different groups (control, cystitis, and carcinoma) were compared by an ANOVA test using the SPSS software Version 29.0.1.0 for Windows. Outliers were identified through graphical representations like boxplots and by calculating the Z score. Both ANOVA tests with and without potential outliers were conducted, and since there was no impact on the results, all data points were included in the analysis. A post hoc Tukey test was performed since the Levene test showed homogeneity of variances. ROC and AUC analyses were performed using the SPSS software for Windows. The statistical significance was set at *p* < 0.05.

## 3. Results

### 3.1. Comparative Quantification of miRNA Isolated from Urine Sediment and Cell-Free Supernatant

For seven control and seven carcinoma cases, the quantification of all ten miRNAs (miR-16, 21, 103b, 106b, 146, 155, 182, 221, 222, and 375) was carried out both on urine supernatants (Table 2) and sediments (Table 3). Six miRNAs (miR-103b, 106b, 146, 155, 182, and 375) were not detectable in the supernatant samples. Four miRNAs (miR-16, 21, 221, and 222) were measurable but at low concentrations in both groups (carcinoma and control). In the urine sediment, miR-103b and 146 were also not measurable. In contrast, the eight other miRNAs were detected at lower (miR-106b, 155, 182, and 375) or higher concentrations (miR-16 and 21).

Since miRNAs in the sediment were measurable at higher concentrations, they were more usable for routine diagnostics; lower values decrease the reliability and sensitivity of detection methods, whereas higher concentrations facilitate consistent quantification, which is essential for diagnostic reproducibility and standardization. Therefore, we focused on the miRNAs found in the sediment.

### 3.2. Quantification of miRNA in Urine Sediment in Cases of Cystitis and UC Compared to Healthy Controls

The analyzed cohort consisted of 18 control samples, 11 cystitis samples, and 18 carcinoma samples. The concentration of copies related to RNU6B (for normalization of cell number) was compared between the three groups.

**Figure 2:**
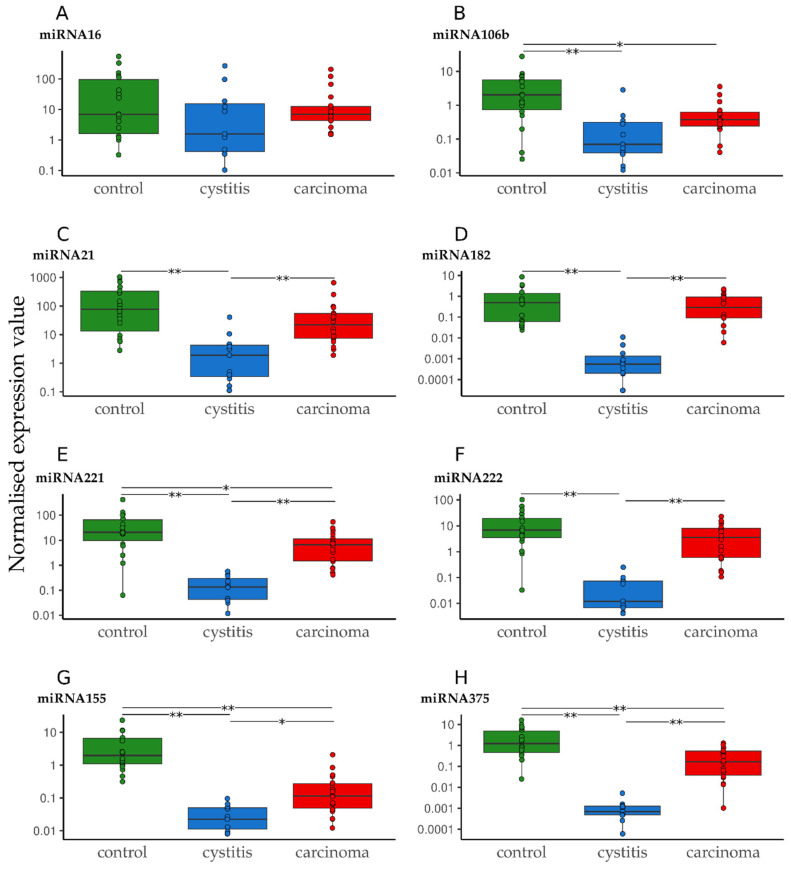
Normalized expression levels of miR-16 (**A**), 106b (**B**), 21 (**C**), 182 (**D**), 221 (**E**), 222 (**F**), 155 (**G**), and 375 (**H**) in different groups (control, cystitis, and carcinoma). The absolute miRNA copy number was related to the internal normalizer RNU6B. The results are shown as normalized expression values with a log 10 scale. * *p* < 0.05, ** *p* < 0.001. Green—control; blue—cystitis; red—carcinoma. The scales are not consistent but adjusted for clearer presentation.

For miR-16, no significant deregulation was seen (Figure 2A, Table 4 and Table S1). MiR-106b was significantly down-regulated in both the cystitis and carcinoma groups compared with the controls (Figure 2B, Table 4 and Table S1). For miR-21, 182, and 222, only the cystitis group showed significant down-regulation compared to the controls and carcinoma groups, respectively (Figure 2C–F, Table 4 and Table S1). Significant deregulation was detected between all three groups for miR-155, 221, and 375 (Figure 2G,H, Table 4 and Table S1).

Furthermore, groups were combined and compared with the third group (Table 4): the combined control/cystitis group showed a significant difference compared to the carcinoma group only for miR-155 and 182. In contrast, the combined cystitis/carcinoma group showed significant deregulation for all except miR-16. The same situation was observed for the combined control/carcinoma group compared with the cystitis group.

### 3.3. Potential of miRNAs as Biomarkers for UC in Dogs

Based on the observed deregulation of some miRNAs between the three groups alone and combined, the potential of these miRNAs as biomarkers was further evaluated using a receiver operating characteristic (ROC) curve analysis. Area under the curve (AUC) values enable the estimation of the power of a predictive model. A value of 0.5 indicates random guessing, whereas a score of 1 indicates perfect performance. Values over 0.8 are considered excellent for differentiation, as both sensitivity and specificity need to be high to achieve such high AUC values.

The AUC values were high (>0.9) for miR-21, 155, 182, 221, 222, and 375 for differentiation between the control and cystitis groups (Figure S1) and for miR-182, 221, 222, and 375 for differentiation between the cystitis and carcinoma groups (Figure S2), respectively. Compared with the carcinoma samples, the combined control/cystitis group showed AUC between 0.454 and 0.651 for all miRNAs (Figure S3), meaning that no discrimination can be achieved in this combination. The combined cystitis/carcinoma group compared to the controls showed high AUC (>0.9) for miR-155 only (Figure S4).

**Figure 3:**
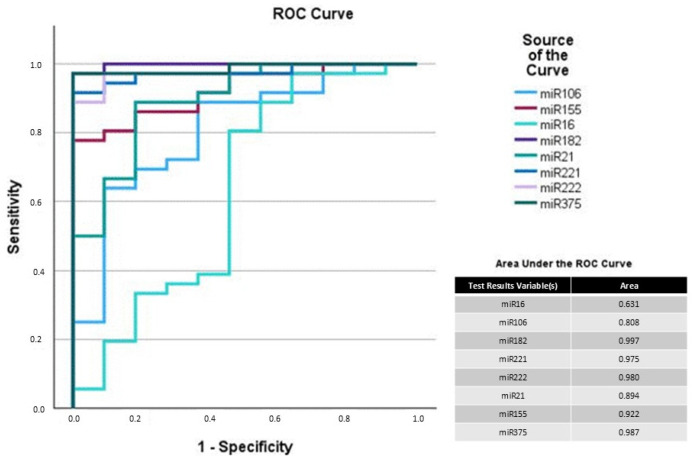
Receiver operating curve (ROC) analysis of analyzed miRNAs with combined control/carcinoma vs. cystitis. Sensitivity is plotted against specificity on a graph. A diagonal line on this graph indicates random guessing. Any points above this line represent good classification. The ideal prediction method is found near the upper left corner, resulting in an area under the curve (AUC) > 0.9.

**Figure 4:**
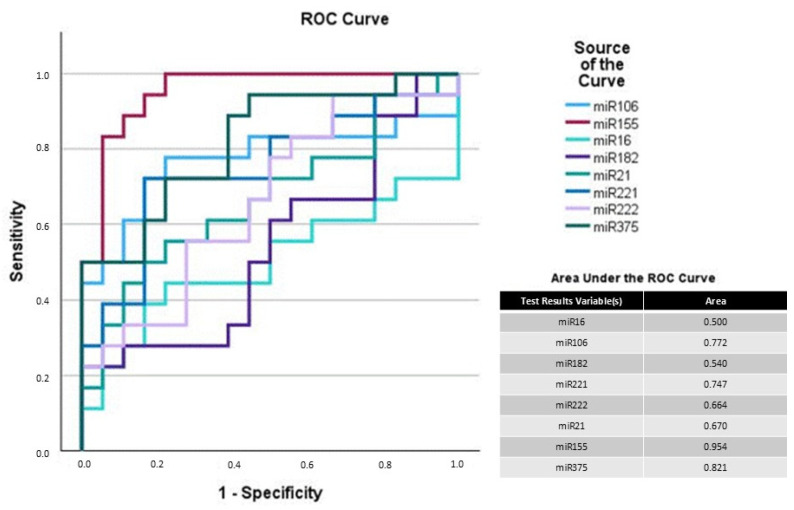
Receiver operating curve (ROC) analysis of analyzed miRNAs with control vs. carcinoma. Sensitivity is plotted against specificity on a graph. A diagonal line on this graph indicates random guessing. Any points above this line represent good classification. The ideal prediction method is found near the upper left corner, resulting in an area under the curve (AUC) > 0.9.

AUC for the cystitis group compared with the control/carcinoma group showed excellent AUC for miR-182 (0.997), 155 (0.922), 221 (0.975), 222 (0.980), and 375 (0.987) (Figure 3). Additionally, miR-155 and 375 reached high AUC values at 0.954 and 0.821 when comparing the control with the carcinoma group (Figure 4). Therefore, a two-step analysis, including as the first step the elimination of cystitis samples and as the second step the discrimination of carcinoma from the remaining control samples, could be a possible approach when using quantification of miR-182 and 155 alone or in combination with miR-221, 222, or 375.

## 4. Discussion

UC is an important health issue in dogs [1]. Dogs would benefit from early diagnosis by non-invasive tests. *BRAF* V595E is already a good biomarker for UC, especially in the Terrier breed [4,6]. But many (about 30%) dogs suffer from UC without having a *BRAF* mutation or copy number alteration, which is the reason for not being diagnosed by the *BRAF* test [4,6,33]. To detect these cases, miRNAs could be possible biomarkers. Additionally, urine seems to be a suitable biological fluid, since it has direct contact with the bladder epithelium and UC, if present [18]. In dogs, a handful of miRNAs were described as deregulated in the urine samples of dogs with UC in comparison with the cystitis and control groups; miR-16 and 103b were down-regulated [18], whereas miR-182, 221, and 222 were up-regulated [19]. In human cases, many miRNAs were described as being deregulated, with miR-21, 146, 155, and 375 appearing multiple times in different studies [20,21,22,23,24,25,26,27,28,29,30,34]. These studies differ in the investigated miRNAs as well as the analyzed urine fraction. Therefore, one central question is which part of the urine is the best for miRNA isolation: supernatant including cell-free miRNAs, sediment including miRNAs in exfoliated cells, or EVs actively being released by cells. This study aimed to determine the best sample material and a suitable miRNA biomarker to detect canine UC in urine in routine diagnostic cases like pre-surgical screening in suspicious cases and/or screening in predisposed breeds.

In many studies, urine supernatant was used for the successful isolation and quantification of miRNA [22,26,27,28,29,30]. Though here only miR-16, 221, 222, and 21 were detectable in low concentrations, and the other miRNAs were not detected at all. Some studies have indicated that miRNAs can be detected and are stable in urine [34,35]. However, there is also evidence suggesting that isolating miRNA from urine supernatants is more challenging than from other biofluids. Factors such as the low abundance of miRNAs and their additional degradation over time can complicate accurate quantification [36]. Additionally, the acidic nature of urine [18,37], along with other components in the supernatant that act as PCR inhibitors [38], can hinder efficient miRNA isolation and amplification. Differences between the miRNAs, especially being bound to proteins or EVs or not, may play a role in detectability in the urine supernatant. In the current study, more miRNAs were detectable from the cells of the urine sediment and at higher concentrations than in the supernatant. This is the first study directly comparing miRNA values isolated from urine supernatants and sediments in dogs, resembling results from a similar study in humans [36].

Since more miRNAs were measurable in higher concentrations when isolated from the urine sediment, we focused on the urine sediment. Subsequently, the miRNAs were compared between dogs with UC, dogs with cystitis, and control dogs.

Only miR-16 showed no significant deregulation between these groups. The expression of miR-16 was described as being higher in canine UC formalin-fixed and paraffin-embedded tissue than in the healthy samples [17], whereas it was not mentioned as deregulated in another study of the miRNome of UC [16]. In the urine samples of dogs with UC, miR-16 was down-regulated [18], whereas it was not mentioned in human studies of urine so far [34].

All remaining miRNAs were significantly down-regulated in the cystitis samples compared to the control and UC samples. A reason for this could be the presence of inflammatory cells (mainly neutrophils, which represent about 90% of the cells) in cystitis urine, but not in control and UC urine. Assuming that the investigated miRNAs are cell-type specific and only expressed in bladder epithelial cells, but not in immune cells, whereas RNU6B is ubiquitously expressed, the ratio between miRNA and RNU6B is lower in cystitis urine solely because of the presence of inflammatory cells. This issue was also discussed as a reason for the differences in miRNA deregulation between the tissue and liquid samples [18]. Measuring neutrophil-specific miRNAs in future studies may strengthen this hypothesis. Nevertheless, the down-regulation of miR-106b was not seen in the urine samples of cystitis dogs in another study [18]. The study investigating miR-182, 221, and 222 included cystitis samples in the control group, and, therefore, the results cannot be directly compared [19]. In nearly all human studies (miR-21, 155, 375), only carcinoma samples were compared to healthy individuals, and no cystitis group was included [20,21,22,23,24,25,28,29]. One study showed the same expression of miR-155 in healthy and cystitis patients (with an up-regulation in carcinoma patients), but they investigated cell-free miRNA in contrast to the urine sediment in our study [30]. It has to be noted that, in contrast to other studies, the character and degree of cystitis were evaluated by cytology. We included only cases with moderate to marked purulent cystitis. Other types (hemorrhagic, mixed cellular) and lower degrees should be evaluated in further studies. In cases of UC, a mild secondary inflammation is commonly present, and an overlap of the miRNA results may appear.

For miR-21, 182, 221, and 222, a significant down-regulation was seen for the cystitis group compared to the control or UC groups, respectively. In a recent study, miR-182, 221, and 222 were described as up-regulated in canine UC compared to a control group consisting of healthy dogs and dogs with cystitis [19]. When combining our control and cystitis dogs in one “non-tumor” group, there was also a trend toward the up-regulation of miR-182 (even significant with *p* = 0.044), 221, and 222 (Table 4). Additionally, miRNAs were isolated from EVs and not from urine sediments. The source of the investigated miRNA seems to play a significant role, since differences between free miRNAs and EV-associated miRNAs were already described in other sample materials and carcinomas [20,39,40]. Differences in study designs could cause the discordance with the finding of up-regulation of miR-21 in human studies: focus on prostate cancer [20], a different quantification strategy using not RNU6B as a normalizer [21], isolation of cell-free and cell-included miRNAs as a whole and using 5S rRNA as a normalizer [22], and—again, as in the canine study mentioned above—miRNA isolation out of EVs [23,24].

For miR-106b, a significant down-regulation was observed for both cystitis and UC compared with the control group. This is in concordance with a recent study describing a down-regulation of miR-106 in UC samples compared to controls, whereas the cystitis samples were not significantly down-regulated, but a similar trend can be seen [18].

For miR-155 and 375, deregulation between all three groups was visible with lowered values in the carcinoma samples and even lower in the cystitis samples. In contrast, in human UC, these two miRNAs were described as up-regulated in carcinoma patients compared with healthy subjects. Again, the source of the miRNA seems to play an important role, since miRNA isolated from the EVs [24,25,28] or supernatants was used [30]. Another study used a totally different analysis method (EXPARs with surface concentration and hybridization to microgels) [29].

In summary, the source of miRNA, the quantification strategy, and the definition of groups can influence the outcome of studies and hamper the comparability of the results. Additionally, distinctions between tissue and urine miRNA profiles make searching for biomarkers more difficult. The relevance of found miRNAs in urine for cancer development is uncertain since they do not seem to reflect the complete miRNA profile of cancer cells.

Suitable biomarkers would detect carcinoma cases in a group that includes healthy, cystitis, and tumor samples. Unfortunately, none of the analyzed miRNAspl herein showed the potential for this. But ROC and AUC analysis suggested possible discrimination between the groups using a two-step analysis: miR-182, 221, or 222 for identifying cystitis; after eliminating the cystitis samples, miR-155 and 375 have the potential to differentiate between the control and carcinoma samples. The corresponding AUC values indicated excellent discrimination between the cystitis, carcinoma, and healthy samples.

Since the number of dogs in the current study was small, the results should be seen as preliminary. Further investigations of more samples could improve the reliability of miR-182, 221, 222, 155, and 375 as a possible biomarker panel for UC. Low-grade cystitis samples should especially be included to make sure that differentiation is possible. False negatives and positives because of overlapping expression ranges must be ruled out. In a further step, cut-off values should be defined as described for miR-122 in liver diseases, for example [41]. It would be interesting to compare the urine miRNA profile of *BRAF*-positive and *BRAF*-negative UC. However, until now, we could not collect a representative number of urine samples from dogs harboring *BRAF*-negative UC.

## 5. Conclusions

In conclusion, the comparison of miRNA copy numbers isolated from canine urine supernatants and sediments showed a more straightforward quantification in urine sediments. Additionally, differences and similarities of urine miRNA profiles between human and canine UC were found. Furthermore, a biomarker panel including miR-155, 182, 221, 222, and 375 has the potential to discriminate among all three groups in a two-step approach.

## Figures and Tables

**Table 1:** Signalment of the dogs included in the study.

Group	Breed	Age (Years)	Sex
Control *n* = 18	1 Beagle, 1 Chihuahua, 1 Dalmatian, 1 German Wirehaired Pointer, 2 Golden Retrievers, 1 Flat-Coated Retriever, 1 Husky, 1 Magyar Vizsla, 1 Malinois, 1 Pug, 1 Tibetan Terrier, 6 mixed breeds	1–15	1f, 5fc, 6m, 5mc, 1u
Cystitis *n* = 11	1 Eurasier, 1 French Bulldog, 1 German Shepherd, 1 Golden Retriever, 1 Labrador Retriever, 1 Magyar Agar, 1 Olde English Bulldogge, 1 Rottweiler, 3 mixed breeds	2–13	4f, 2fc, 1m, 4mc
Carcinoma *n* = 18	1 Border Collie, 1 Boxer, 1 Bullmastiff, 1 English Bulldog, 1 German Spitz, 2 Jack Russell Terriers, 1 Magyar Vizsla, 1 Pug, 1 Scottish Terrier, 1 Shih Tzu, 7 mixed breeds	6–13	3f, 7fc, 4m, 4mc

f = female, fc = female castrated, m = male, mc = male castrated, u = unknown.

**Table 2:** Concentration in copies/µL of ten different miRNAs isolated from the urine supernatant from dogs with and without UC.

miRNA	Controls (*n* = 7)		Carcinoma (*n* = 7)	
	Median	Range	Median	Range
16	0.9	0–16.3	0.49	0–12.8
21	11.8	0–48.6	11.2	1.15–185
103b	0		0	
106b	0		0	
146	0		0	
155	0		0	
182	0		0	
221	0.9	0–16.7	5.5	0.19–29.5
222	1.01	0–2.8	1.19	0–1.7
375	0		0	

**Table 3:** Normalized miRNA values (copies related to the standard U6) of ten different miRNAs isolated from the urine sediment from dogs with and without UC.

miRNA	Controls (*n* = 7)		Carcinoma (*n* = 7)	
	Median	Range	Median	Range
16	124.1	24–548	10.5	1.6–121
21	343.1	45–1047	51.1	3–253
103b	0		0	
106b	6.9	0.5–28	0.6	0.1–4
146	0		0	
155	5.5	0.7–23	0.2	0.1–0.8
182	3.3	0.1–8	0.5	0.1–1.3
221	72.2	18–418	5.8	0.8–54
222	28.5	7–102	2.9	0.1–23
375	3.8	0.6–10	0.5	0.1–1.3

**Table 4:** *p*-values of ANOVA and post hoc Tukey test analysis of different groups, single and combined.

	Tukey			ANOVA		
	Control vs. Cystitis	Control vs. Carcinoma	Cystitis vs. Carcinoma	Control/Cystitis vs. Carcinoma	Control vs. Cystitis/Carcinoma	Control/Carcinoma vs. Cystitis
miR-16	0.210	0.896	0.380	0.763	0.274	0.095
miR-21	<0.001	0.167	<0.001	0.655	0.002	<0.001
miR-106b	<0.001	0.030	0.067	0.506	<0.001	<0.001
miR-155	<0.001	<0.001	0.005	0.042	<0.001	<0.001
miR-182	<0.001	0.727	<0.001	0.044	0.004	<0.001
miR-221	<0.001	0.049	<0.001	0.525	<0.001	<0.001
miR-222	<0.001	0.172	<0.001	0.256	<0.001	<0.001
miR-375	<0.001	<0.001	<0.001	0.691	<0.001	<0.001

## Data Availability

Data contained within this article and Supplementary Materials.

## Supplementary Materials

The following supporting information can be downloaded at https://www.mdpi.com/article/10.3390/vetsci12070621/s1, Figure S1: Receiver operating curve (ROC) analysis of analyzed miRNAs with control vs. cystitis; Figure S2: Receiver operating curve (ROC) analysis of analyzed miRNAs with carcinoma vs. cystitis; Figure S3: Receiver operating curve (ROC) analysis of analyzed miRNAs with combined control/cystitis vs. carcinoma; Figure S4: Receiver operating curve (ROC) analysis of analyzed miRNAs with combined cystitis/carcinoma vs. control; Table S1: miRNA copy values (isolated from urine sediment) related to RNU6B, log transformed.
