# Supplementary figures and images for: Utility of Urinary miRNA Biomarkers for Canine Urothelial Carcinoma Diagnostics
Source: Vet Sci. 2025 Jun 27;12(7):621. doi: 10.3390/vetsci12070621 (PMC12298497; doi:10.3390/vetsci12070621)

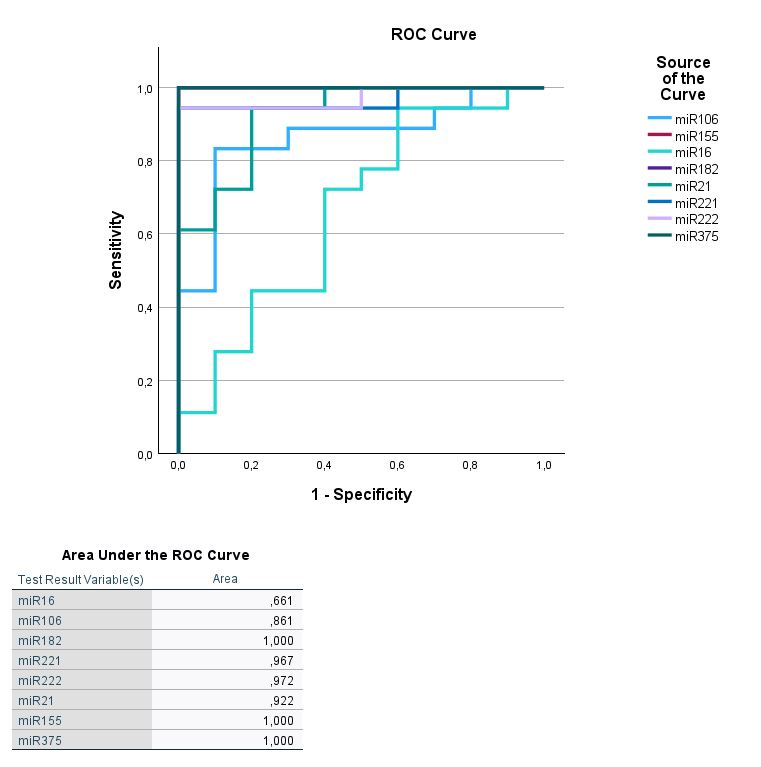

Supplement: Supplementary file 1 [file vetsci-12-00621-s001.zip › Figure S1.JPG]

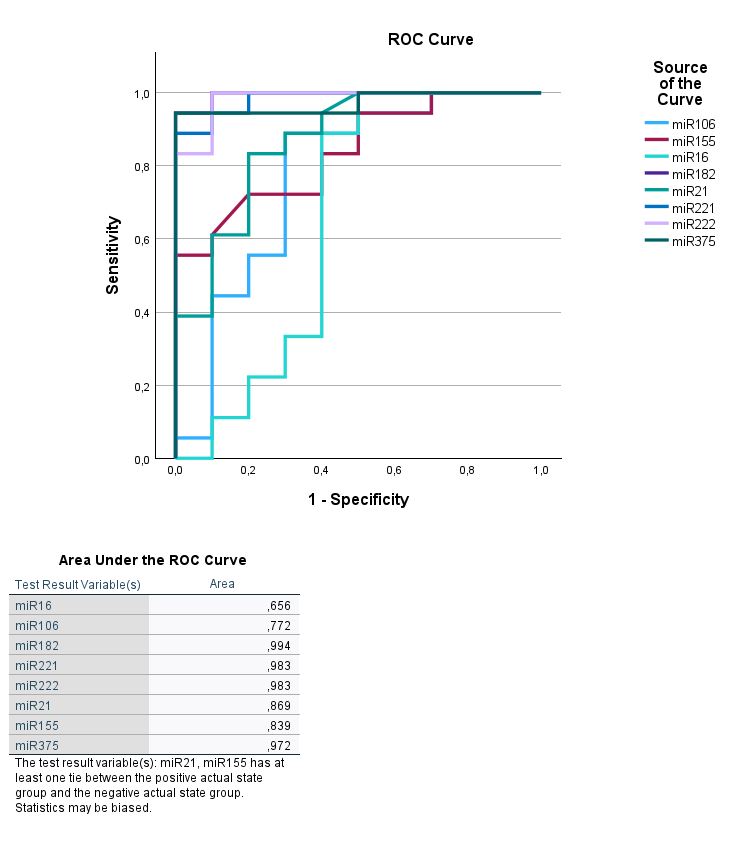

Supplement: Supplementary file 1 [file vetsci-12-00621-s001.zip › Figure S2.JPG]

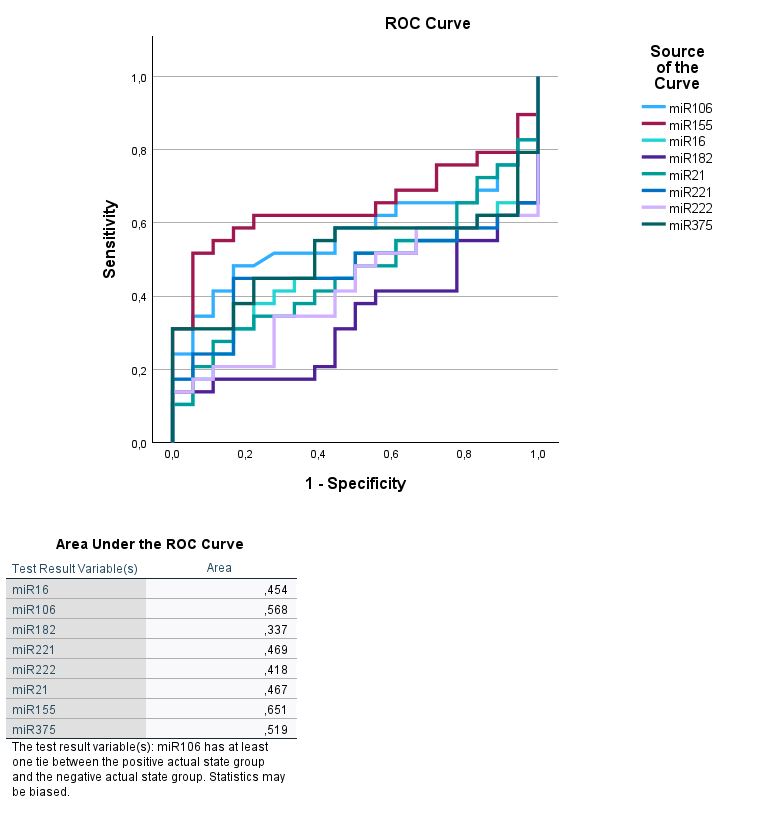

Supplement: Supplementary file 1 [file vetsci-12-00621-s001.zip › Figure S3.JPG]

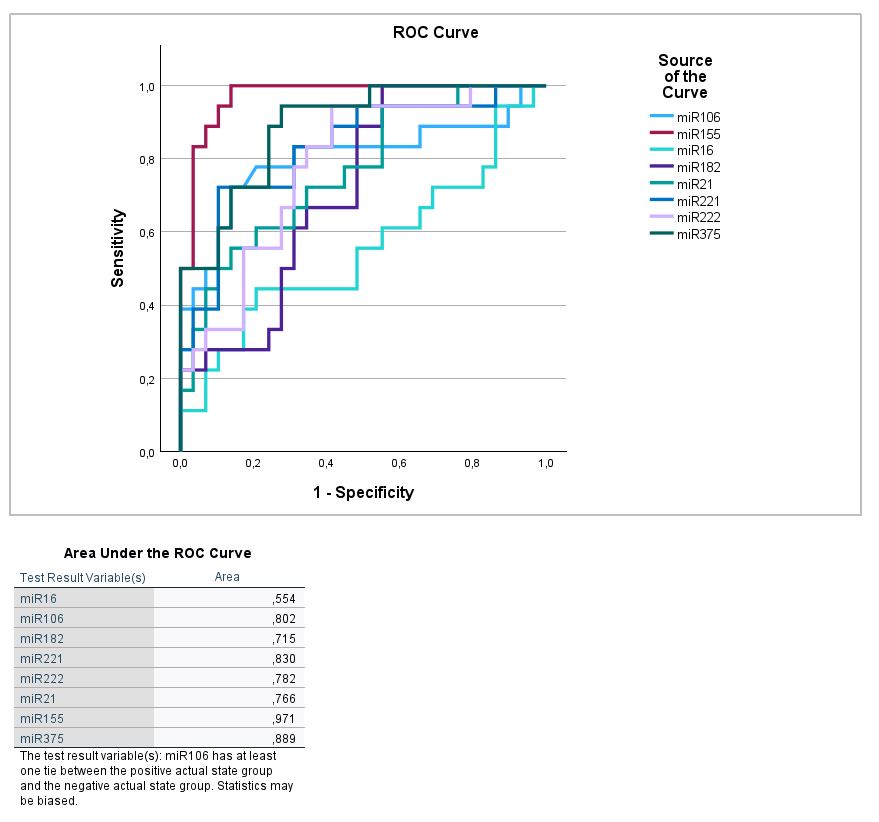

Supplement: Supplementary file 1 [file vetsci-12-00621-s001.zip › Figure S4.JPG]
